# A Multiscale Deep‐Learning Model for Atom Identification from Low‐Signal‐to‐Noise‐Ratio Transmission Electron Microscopy Images

**DOI:** 10.1002/smsc.202300031

**Published:** 2023-06-11

**Authors:** Yanyu Lin, Zhangyuan Yan, Chi Shing Tsang, Lok Wing Wong, Xiaodong Zheng, Fangyuan Zheng, Jiong Zhao, Ke Chen

**Affiliations:** ^1^ School of Future Technology South China University of Technology Guangzhou 510641 China; ^2^ Department of Applied Physics The Hong Kong Polytechnic University Kowloon Hong Kong China; ^3^ Polytechnic University of Hong Kong Shenzhen Research Institute Shenzhen 518057 China; ^4^ Peng Cheng Laboratory Shenzhen 518055 China; ^5^ School of Electronic and Information Engineering South China University of Technology Guangzhou 510641 China

**Keywords:** atomic positions, image segmentations, supervised learning, transition metal dichalcogenides, U-Net

## Abstract

Recent advancements in transmission electron microscopy (TEM) have enabled the study of atomic structures of materials at unprecedented scales as small as tens of picometers (pm). However, accurately detecting atomic positions from TEM images remains a challenging task. Traditional Gaussian fitting and peak‐finding algorithms are effective under ideal conditions but perform poorly on images with strong background noise or contamination areas (shown as ultrabright or ultradark contrasts). Moreover, these traditional algorithms require parameter tuning for different magnifications. To overcome these challenges, AtomID‐Net is presented, a deep neural network model for atomic detection from multiscale low‐SNR experimental images of scanning TEM (scanning transmission electron microscopy (STEM)). The model is trained on real images, which allows the robust and efficient detection of atomic positions, even in the presence of background noise and contamination. The evaluation on a test set of 50 images with a resolution of 800 × 800 yields an average F1‐Score of 0.964, which demonstrates significant improvements over existing peak‐finding algorithms.

## Graphical abstract

AtomID‐Net is an advanced deep‐learning network designed for rapid detection of atomic locations in scanning transmission electron microscopy low‐signal‐to‐noise‐ratio high‐angle annular dark‐field images. It leverages a robust dataset of real‐world atomic columns, enabling accurate and efficient identification of atomic positions.

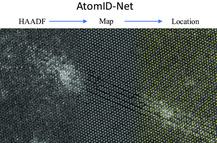

## 1 Introduction

Electron microscopy (EM) data with atomic resolution contain massive information of spatial distribution of atomic and electronic structures.^[^ ^1^, ^2^ ^]^ The primary step of structural analysis based on high‐resolution (HR) transmission electron microscopy (TEM) is to quantitatively correlate the local intensity peaks/valleys with the atoms/atomic columns in samples. Accurate understanding and engineering on the atomic structures is essential for various materials.^[^ ^3^ ^]^ With the aberration correctors developed for TEM in recent years,^[^ ^4^, ^5^, ^6^ ^]^ scale of the TEM images can be down to the tens of picometers(pm). Particularly, atomic structural information can be readily explained using the scanning TEM (STEM) techniques.^[^ ^7^, ^8^, ^9^, ^10^ ^]^

Herein, we applied the HRSTEM imaging technique for the structural analysis on the 2D materials, such as 2D transition metal dichalcogenides (TMD).^[^ ^11^, ^12^, ^13^, ^14^ ^]^ These materials exhibit strong photomaterial coupling and photoelectric properties, making them suitable for next‐generation optoelectronic devices.^[^ ^15^, ^16^ ^]^ However, due to their ultrathin atomic thickness, a slight change in atomic structures may cause great impacts on their physical properties. For example, ReS_2_, a new member of 2D TMD materials, exhibits anisotropic electrical conductivity behavior due to the distorted 1T phase structure. In monolayer (1L) ReS_2_, the bandgaps vary from 1.3 to 1.8 eV due to different types of grain boundaries.^[^ ^17^ ^]^ The conductance of ReS_2_ will also change due to different phase structures.^[^ ^18^ ^]^ In addition, the presence of Re vacancies regulates the electronic structure as well as the electrochemical performances,^[^ ^19^ ^]^ and the generation of a single‐atom defect in Re leads to a locally spin‐polarized ground state with magnetic moments of 1–3 *μ*_B_.^[^ ^20^ ^]^ Therefore, clarification of structural information is highly important to understand the structure–property relationships in these materials.

2D materials are ideal samples for HRTEM/STEM characterizations, because each atomic position in the 2D layers can be easily distinguished from a single TEM/STEM image. However, experimental acquisition of high‐quality atomic‐scale TEM/STEM images requires high‐end TEM facility and extensive professional expertise in electron optics for the TEM operators. Furthermore, environmental disturbances such as air flow, noise, vibration, AC and DC magnetic fields, and temperature fluctuations could introduce additional nonuniform noise in TEM images, which will dramatically reduce the quality.^[^ ^21^ ^]^ By increasing the electron dose for imaging, the resolution and signal‐to‐noise ratio (SNR) of TEM/STEM images could be improved if the mechanical stabilization of the TEM instrument is well maintained. However, many materials including 2D materials are beam sensitive and vulnerable to irradiation damage.^[^ ^22^ ^]^ In real TEM/STEM experiments, the low‐dose imaging condition is often required, which precludes the possibility to capture high‐quality images. On the other hand, using low accelerating voltage of TEM can reduce the knock‐on damage on samples.^[^ ^23^, ^24^, ^25^ ^]^ However, the spatial resolution is also greatly compromised under low voltage.^[^ ^26^ ^]^ Therefore, atomic structure analysis based on the nonideal, unclear TEM/STEM images is of high importance and pressing for the field.

Another significant challenge is the contamination caused by the spontaneous adsorption of hydrocarbon species inside the TEM chamber on the sample surfaces during STEM imaging.^[^ ^27^, ^28^ ^]^ Due to the ultrathin thickness of samples and the resulting ultralow contrast of STEM signals, hydrocarbon contamination could appear as ultrabright/dark spots/zones, adding noisy backgrounds that obscure the real sample information.

Currently, conventional analysis on low‐SNR STEM images rely on filters, such as the Wiener filter^[^ ^29^ ^]^ for denoising. However, filters based on strict criteria^[^ ^30^ ^]^ only work well on periodic samples. The atomic defects, dopants, surfaces, and other nonperiodic structures may deviate from the original contrast intensity and atomic position after filtering. While existing methods based on Gaussian template matching can preserve original structures, they lack dynamic and multiscale adaptability.^[^ ^31^ ^]^ Conventional algorithms require tedious manual operations, and the accuracy is not always satisfactory. Moreover, researchers often base their adjustments and operations on subjective feelings when they lack quantified performance metrics. Therefore, an efficient and convenient method for the automatic detection of atomic positions and columns, along with a reliable evaluation protocol, needs to be established to address these issues.

We present a new method for automatic detection of atomic positions and columns in STEM images using powerful deep networks. Our method does not rely on explicit prior filtering, reducing intrinsic errors and improving atomic structure interpretation. Unlike traditional template matching methods,^[^ ^32^ ^]^ our model learns features from a large number of automatically labeled STEM images of real‐world samples with known and compatible structures, enabling it to distinguish known atomic structures and the noise in the STEM images implicitly. Additionally, our method can locate foci in parallel across multiple scales, providing faster and more efficient performance than existing template‐based methods.

We compared our method with the previous works, including Lee et al.,^[^ ^33^ ^]^ Madsen et al.,^[^ ^34^ ^]^ Lin et al.,^[^ ^35^ ^]^ and Yang et al.^[^ ^36^ ^]^ These approaches used variations of the U‐Net^[^ ^37^ ^]^ architecture and additional techniques to improve denoising performance, but ultimately relied on template matching algorithms at the final detection stage. Inspired by another cell detection work Stardist,^[^ ^38^ ^]^ our approach outperforms these methods and does not require a fixed scale of atoms. Additionally, we introduce a new data labeling method that enables us to quickly and accurately label noisy images.

Our work makes several contributions, including: 1) a new and accurate labeling method, 2) a deep‐learning network for atom detection from low‐SNR STEM image, and 3) a set of benchmarking metrics on atom detection tasks.

## 2 Models And Methods

### 2.1 Architecture

The AtomID‐Net architecture used in this study is based on the popular U‐Net encoder–decoder structure, originally designed for image segmentation. In this article, the U‐Net architecture is adapted for the task of atomic segmentation. The model takes in TEM images as inputs and their corresponding semantic labels as targets to train the model.

The AtomID‐Net architecture consists of convolutional blocks with 3 × 3‐kernel and proper skip links. Each convolutional block has two convolutional layers and two rectified linear units (ReLU). Skip links map outputs from the blocks of the encoder to the corresponding blocks of the decoder. Instead of the original linear layers, a convolutional block is used at the bottleneck, and an extra branch is designed to predict the global average atomic spacing.

The main branch for segmentation is parameterized by *θ* and denoted by $f_{\theta }\left(\cdot \right)$, while the extra branch for spacing prediction is parameterized by *μ* and denoted by $g_{\mu }\left(\cdot \right)$. Although *θ* and *μ* partially overlap in the encoder, the side effect of the partial merge is ignored in training.

The training set consists of input image set ${\left\{X_{i}\right\}}_{i=1}^{N}$ and the corresponding target image set ${\left\{Y_{i}\right\}}_{i=1}^{N}$. The targets are images with annotations at the foci of the atoms from the inputs. The *i*‐th target image contains $n_{i}$ annotated centers. An assumption is made that every observable atom has a shape of an approximate disc, and its geometric center gives a local maxima of intensity. All locations of atoms in the *i*‐th target image can be constructed as

(1) $$Y_{i}=\;\sum_{y\in {\mathbb{R}}^{2}}\delta \left(y\right)$$

where $Y_{i}$ denotes the *i*‐th dot‐annotated label image, and *y* is the coordinate vector. The δ(y) is a unit impulse function. Then we apply scaled Gaussian filters on all the $n_{i}$ centers; it can be conducted in a convolution operation.

(2) $${\tilde{Y}}_{i}=Y_{i}⊗e^{-\frac{y^{T}y}{2\sigma_{i}^{2}}}$$

(3) $$\sigma_{i}\propto {\bar{s}}_{i}$$

(4)

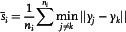

where the global spacing ${\bar{s}}_{i}$ is the mean of all distances of all atoms to their closest counterparts in the *i*‐th sample and the parameter $\sigma_{i}$ is a variance factor proportional to ${\bar{s}}_{i}$. ⊗ denotes a 2D convolution operation; with the kernels it smoothens the labels by turning hollow images with sparse dots into confidence maps. ${\tilde{Y}}_{i}$ represents the *i*‐th smoothed confidence map. The coefficient of the Gaussian kernel is dropped to make a scaled version of the Gaussian filter. It avoids fluctuations of intensity with the spacing varies and enables subsequent operations to favor the centers equally likely.

The task of the main branch is to segment atoms from the input images and output confidence maps that are close to the given target maps. Each output pixel's intensity represents the possibility of being an atomic center. The higher intensity, the closer to the nearest center. The optimal target can be written as
(5)

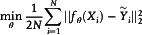

where the *X* is the input of network $f_{\theta }$ with learnable parameters *θ* and ${\tilde{Y}}_{i}$ is the smoothed target confidence map (generated by (2)). The second task is to predict global spacing ${\bar{s}}_{i}$ through another branch $g_{\mu }$. The optimal target can similarly be written as
(6)

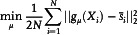




The two target functions are combined with a tradeoff constant to balance training of the both terms, resulting in total loss function *L*

(7)

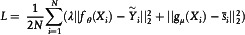




**Figure 1:**
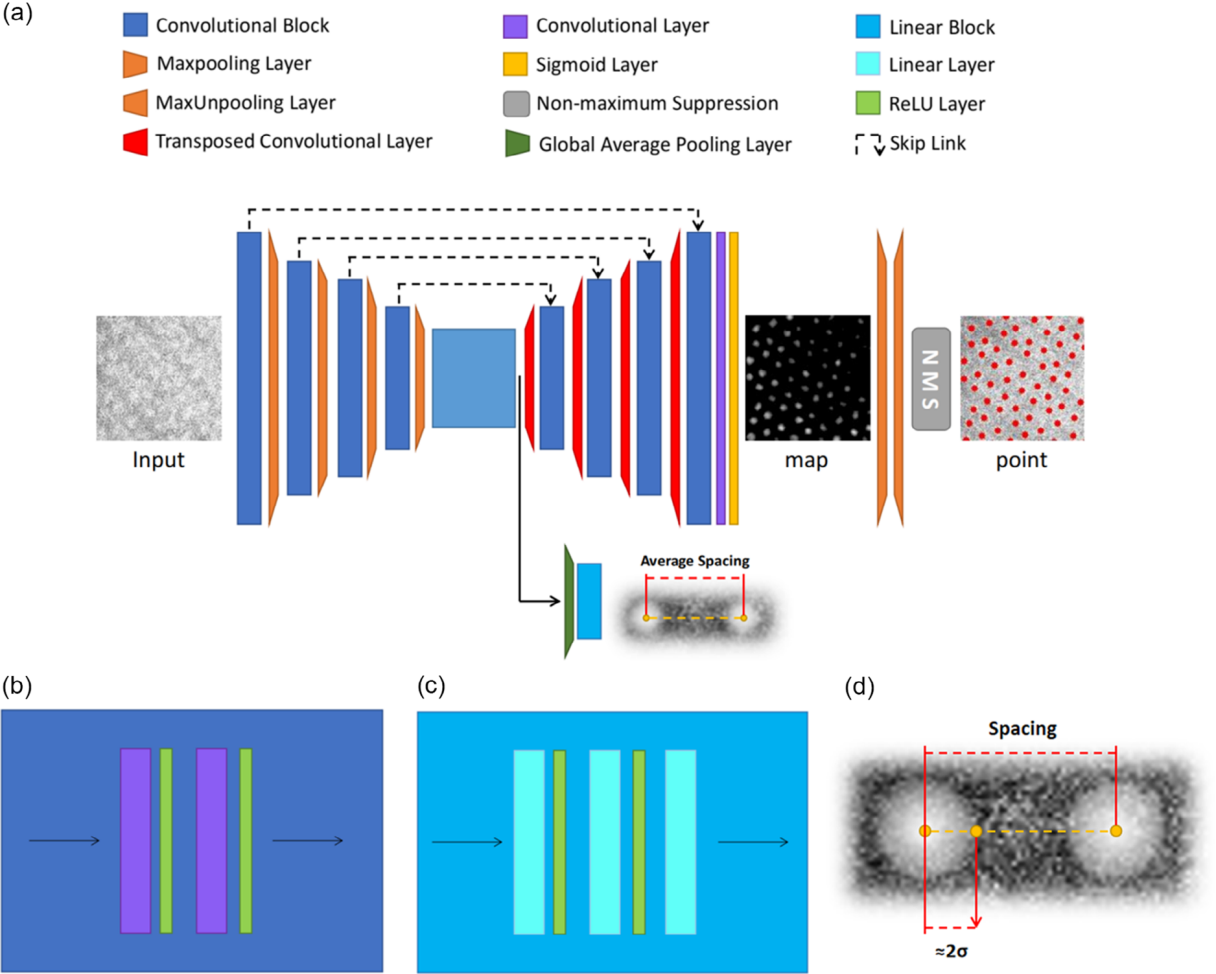
Network architecture of our model. a) Overall schematic architecture of the AtomID‐Net. The main branch outputs the confidence maps of atomic columns. b) Schematic structure of a convolutional block. c) Schematic of a linear block and d) Gaussian kernel assumption approach toward segmenting atoms (see text for more details).

During the detection stage, the Maxpooling and Maxunpooling layers downsample a predicted confidence map and then upsample it back to reduce redundant local maximas and noises. The remaining local maximas are considered as detection proposals and the predicted atomic spacing as the side lengths of detection bounding boxes. These proposals and spacings are sent to the final detection module, nonmaximum suppression (NMS).^[^ ^39^ ^]^ The NMS algorithm finds the most possible atomic centers among the proposals by filtering out those which fail the IOU restrictions and outputs the coordinates. Comparing to other matching algorithms, the time consumption is resolution irrelevant when the count of filtered proposals is fixed. The architecture of the AtomID‐Net model is displayed in **Figure** **1**.

The AtomID‐Net model was trained at an initial learning rate of 10^−3^ on a single graphics processing unit (GPU) 1080Ti for 200 epochs. The learning rate decay to 0.1 times each 50 epochs. *λ* in loss function is set to 0.1. The IOU allow rate in the NMS algorithm is set to 0.3.

### 2.2 Dataset

Real STEM high‐angle‐annular dark‐field (HAADF) data (Figure S1, Supporting Information) were taken from laboratory and split into training set and testing set. The dataset contains 162 images in size of 1024 × 1024 and 50 images in size of 800 × 800 for training and testing, which mainly consists of 1L ${\text{ReS}}_{2}$ of phase $T^{\prime \prime }$ and ${\text{MoS}}_{2}$ of phase *H*. Considering a high cost and unavoidable ambiguity of manual annotation on real images that contain thousands of atoms in each, the training set was labeled automatically by Findfoci.^[^ ^40^ ^]^ For diversity of training data, augmentation such as random resizing, cropping, and additional noise adding were conducted, which thus lead to a big amount of training images.

**Figure 2:**
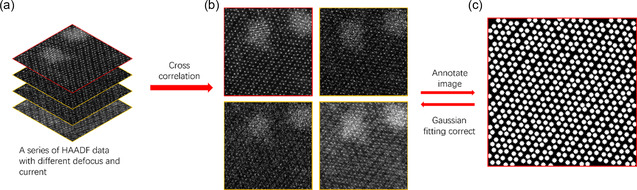
The annotation procedure for test set. a) A group of HAADF images with different defocus and current. b) The cross‐correlation method corrected the image drift. c) The image with the best quality was selected (the image with the red border) and annotated by Findfoci as a reference for the group, and the 2D Gaussian fitting is used to refine the reference into precise labels. (See text for more details).

The testing set was generated using a different approach. Each real‐world sample was imaged 10 times by STEM in a fixed region, with random variations made to the current, defocus, and other parameters of the STEM to capture images under diverse conditions. During this process, drift and spatial deviation were inevitable, resulting in positional differences among atomic positions in all spots. To mitigate drift, the cross‐correlation method was used to align images within each group of shots. To refine labels for each shot, the image with the best visibility was automatically annotated by Findfoci as a reference,^[^ ^40^ ^]^ and then a 2D Gaussian fitting method^[^ ^41^ ^]^ was applied to fine tune the reference and generate precise labels. Visually duplicated samples out of 30 groups were removed, leaving 50 images as the final test set. Specifically, sulfur atoms, which were not dominant and barely observable in the samples, were ignored during the annotation process. The annotation procedure for the test set is illustrated in **Figure** **2**.

### 2.3 Evaluation Method

To evaluate the performance of our AtomID‐Net, we employed three metrics: Chamfer Distance (CD), Jaccard score, and F1 score.

#### 2.3.1 Chamfer Distance

The CD is a measure of the distance between two sets. It describes the expected distance between the prediction *P* with $n_{p}$ centers and the ground truth *T* with $n_{t}$ centers in this study. The algorithm is defined as

(8) $$CD=\frac{1}{n_{p}}\sum_{x_{j}\in P}\underset{y_{k}\in T}{\min}||x_{j}-y_{k}||+\frac{1}{n_{t}}\sum_{y_{k}\in T}\underset{x_{j}\in P}{\min}||x_{j}-y_{k}||$$

where $\;x_{j}$ and $y_{k}$ represent the coordinates of centers from the prediction set *P* and the ground truth set *T* respectively.

#### 2.3.2 Jaccard Score

The Jaccard score is a measure of the overlap between the two sets and is calculated as

(9) $$J\left(P,\;T\right)=\;\frac{\left|P\;\cap \;T\right|}{\left|P\;\cup \;T\right|}$$

(10) $$P\cap T=\{x_{j}\;|\;k\left(j\right)=\underset{k}{\text{argmin}}||x_{j}-y_{k}||,\;j=\underset{h}{\text{argmin}}||x_{h}-y_{k\left(j\right)}||,\;x\in P,\;y\in T\}$$

where *P* is the prediction set, *T* is the ground truth set, and |⋅| calculates the count of points. The intersection ∩ of two sets in these equations is the set of points that pair up, but distances between the partners can be unstable at the edge of an image. The union ∪ of the sets counts every matched pair only once and adds up all the mismatched points from both sets. The score of 0 indicates no overlap, while a score of 1 indicates that two sets are bijective.

#### 2.3.3 F1 Score

The F1 score is a harmonic mean of the precision and the recall and combines them in a single metric, given by

(11) $$\text{precision}=\;\frac{\left|P\;\cap \;T\right|}{\left|T\right|}$$

(12) $$\text{recall}=\;\frac{\left|P\;\cap \;T\right|}{\left|P\right|}$$

(13) $$F_{1}=2\text{* }\frac{\text{precision*recall}}{\text{precision}+\text{recall}}$$

## 3 Results and Discussion

### 3.1 Quality Assessment

**Figure 3:**
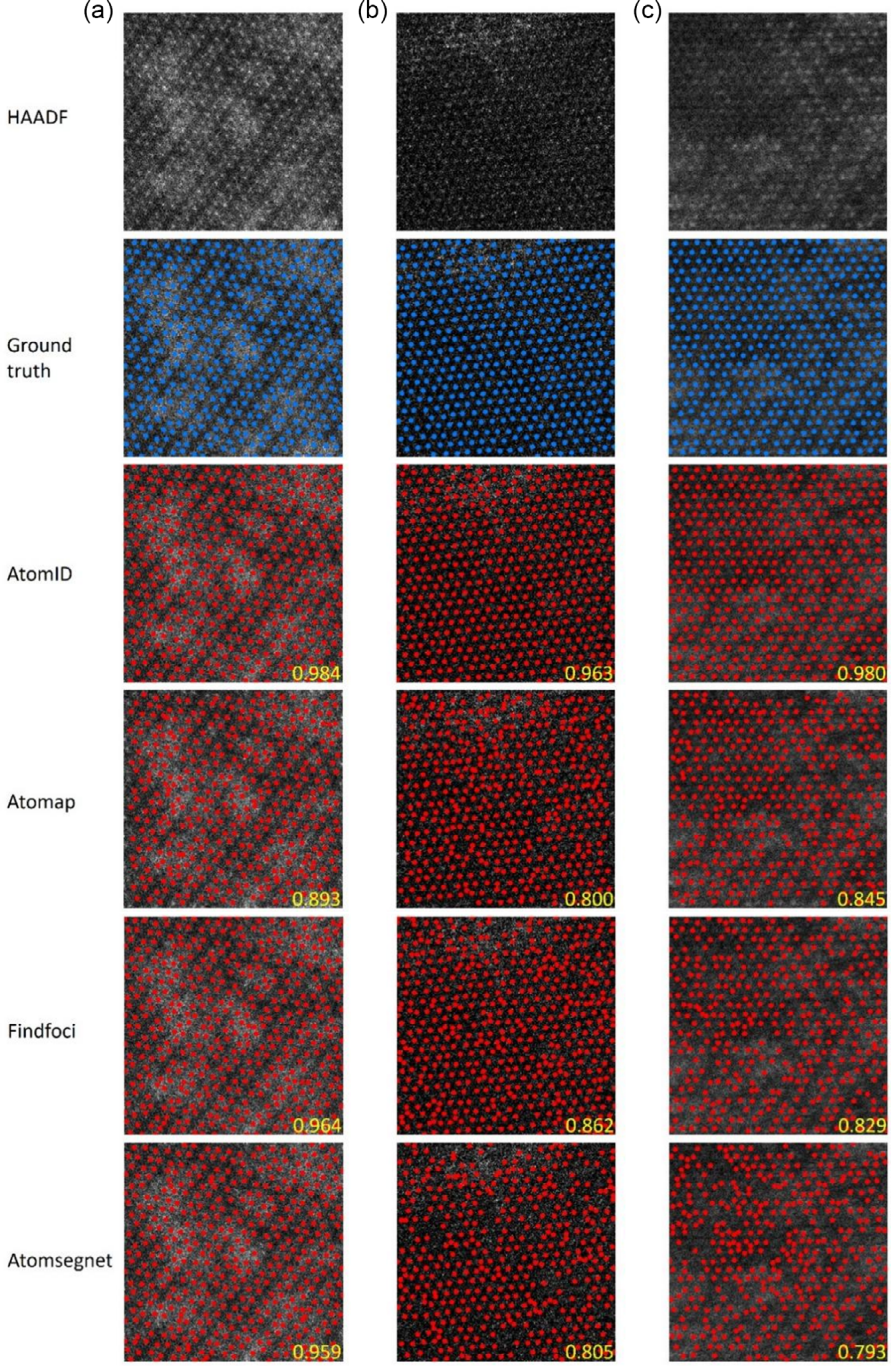
A few examples with different F1‐Scores from the test set. The F1 score is shown in yellow. Atomic scale: a) 6.35 × 6.35 nm; b) 6.35 × 6.35 nm; c) 7.03 × 7.03 nm.

To evaluate the performance of our model, we compared our model with three other methods, Findfoci,^[^ ^40^ ^]^ Atomap,^[^ ^31^ ^]^ and AtomSegNet.^[^ ^35^ ^]^ Findfoci^[^ ^40^ ^]^ is a peak‐seeking algorithm, which searches locally for maximal intensity with a set of moving windows and then expands by gradually allocating the points with lower intensity to the peak region above it. By altering parameters, redundant or false peaks can be merged to improve the performance on noisy images. Labeled images were collected to train this algorithm to select the optimal combination of parameters for batch processing of images. Atomap^[^ ^31^ ^]^ finds all the aligned atomic columns and apply 2D elliptical Gaussian fitting on each column to locate each atom. The constraint of atom alignment tends to give a structurally regularized output. By adjusting the value of the pixel separation, it can be used to identify atomic columns in pictures of different structures and magnifications. AtomSegNet^[^ ^35^ ^]^ was proposed to exploit augment synthetic training images by adding background and Poisson noises to improve robustness against noises. The details of parameters setting are shown in the Supplementary note 1, Supporting Information. In the test of 50 STEM images, the average F1‐Score of the AtomID‐Net model is 0.964, which exceeds the other three methods (**Table** **1**). Note that all methods perform well for clear images, but our model performs better for low‐quality images (**Figure** **3**).

**Table 1:** Quality assessment of different methods

	AtomID	Atomap	Findfoci	AtomSegNet
CD	2.373 ± 1.176	4.174 ± 1.176	2.636 ± 1.176	4.550 ± 1.176
Jaccard	0.967 ± 0.025	0.774 ± 0.025	0.814 ± 0.025	0.733 ± 0.025
F1	0.964 ± 0.039	0.864 ± 0.039	0.891 ± 0.039	0.832 ± 0.039

### 3.2 Scale Assessment

**Figure 4:**
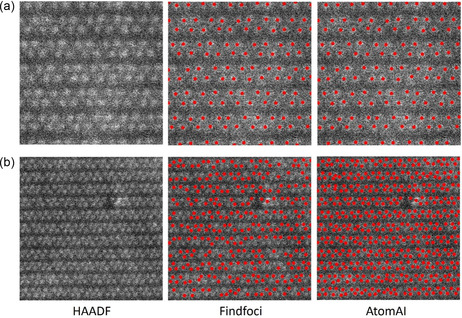
Comparison with Findfoci in different magnification images. Atomic scale: a) 3.75 × 3.75 nm; b) 7.03 × 7.03 nm.

To test the performance of our model at different scales, we used a series of HAADF data with different magnifications to do test, and we compared our model with Findfoci, a peak‐seeking algorithm which we used to generate autoannotated training samples. In comparison, we used HAADF images with different magnifications of the same structure and applied the same set of parameters to Findfoci method and our model. The results in **Figure** **4** show that, when testing on inconsistent scales across images, Findfoci searches exhaustively for optimal hyperparameters to achieve satisfactory results, while our proposed model can address this issue faster, making it more practical (Figure S2, Supporting Information).

### 3.3 Failure Case

**Figure 5:**
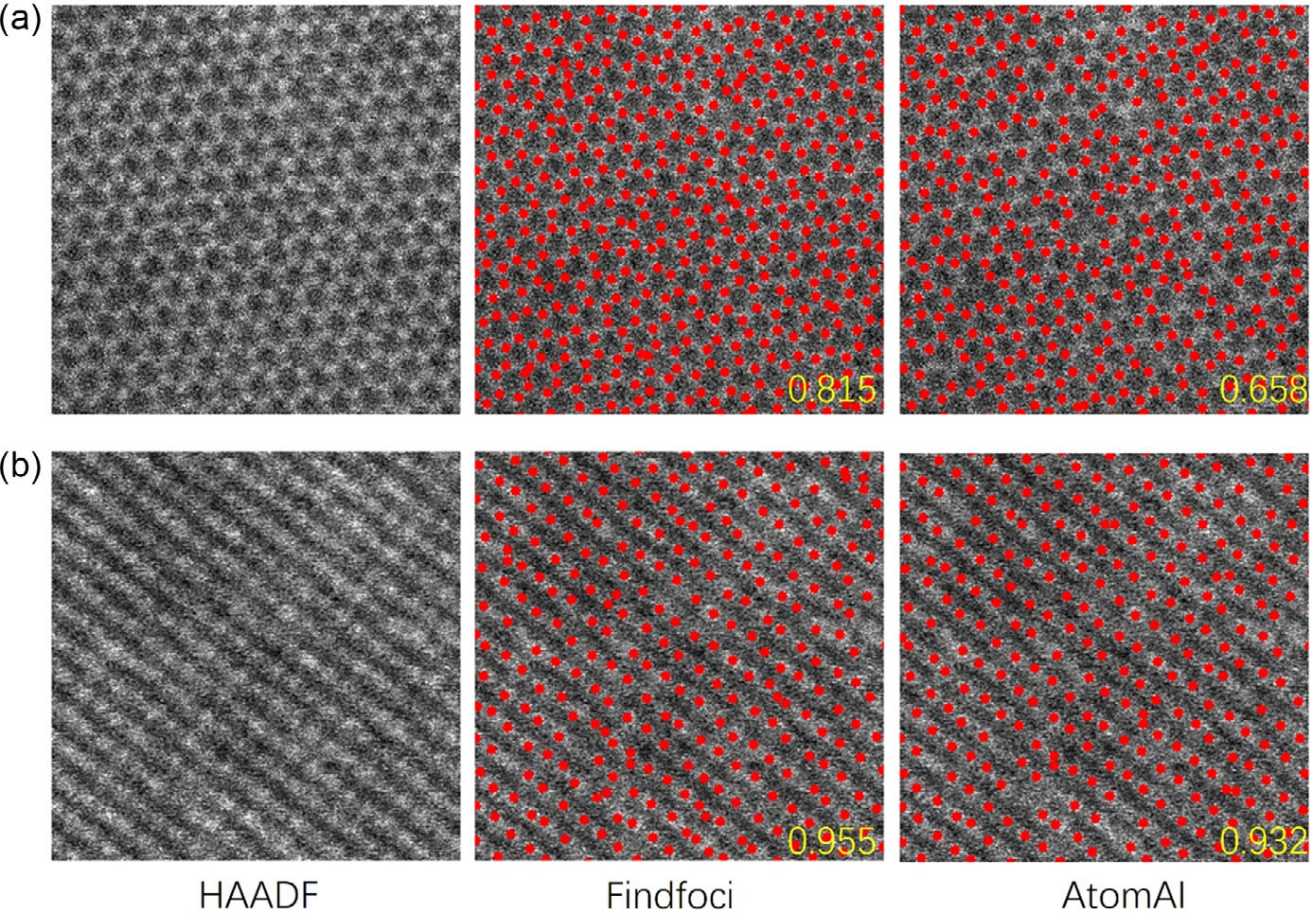
Detection on outliers. The F1‐Score is shown in yellow. a) HAADF image of bilayer MoS_2_. b) HAADF image of BiSeO. Atomic scale: a) 3.18 × 3.18 nm; b) 3.18 × 3.18 nm.

Training and testing on outliers with other structures or nonperiodic materials were also employed in this study. It is concluded that prior knowledge is needed to deal with low‐SNR samples. A model trained only on data of a certain phase has a dramatic bias. When running tests on samples with hexagonal structures (bilayer MoS_2_), it tends to give a detection result satisfying the phase that is trained on, as shown in **Figure** **5**. In our dataset consisting of monolayer ${\text{ReS}}_{2}$ of phase $T^{\prime \prime }$ and ${\text{MoS}}_{2}$ of phase *H*, the sulfur atoms are mostly hidden in noisy backgrounds and has been ignored. However, in the structure of hexagon, atoms in the ignored positions are dominant and are expected to be located. Consequently, training with multiple incompatible structures in a shared pipeline causes conflicts and reduces performance.

### 3.4 Strain Mapping

**Figure 6:**
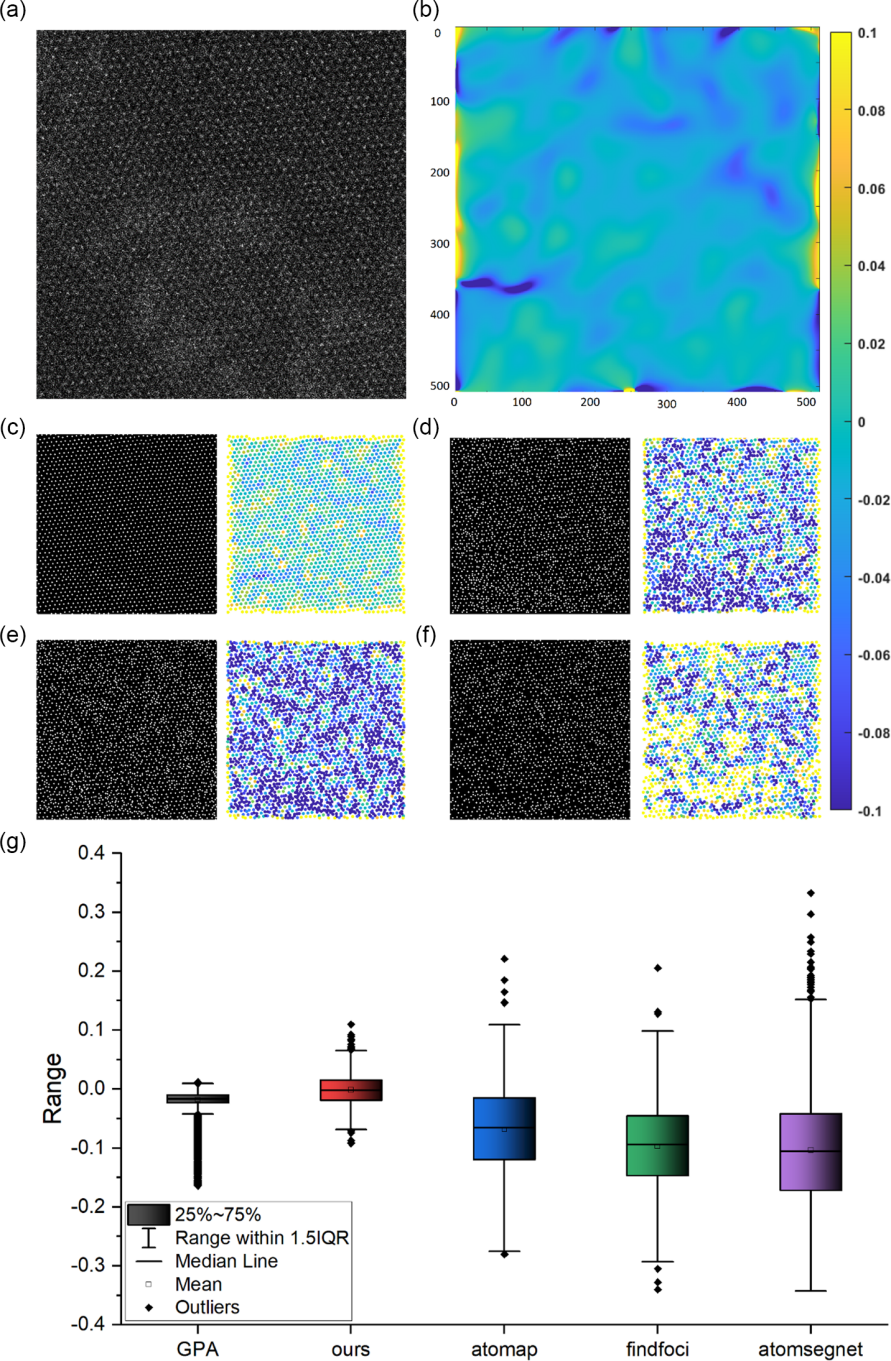
a) Test image. Atomic scale: 12.7 × 12.7 nm. b) Normal strain mapping calculated by GPA method. c) Identification result and normal strain mapping calculated by AtomID. d) Identification result and normal strain mapping calculated by Atomap. e) Identification result and normal strain mapping calculated by Findfoci. f) Identification result and normal strain mapping calculated by AtomSegNet. g) The distribution range of the five strain mappings above.

To further evaluate our model's performance, we compared it with other methods, including Findfoci, Atomap, and AtomSegNet, using them to locate the atomic columns and calculate the strain maps. We also used the result of geometric phase analysis (GPA) method^[^ ^42^ ^]^ as a reference image for 2D strain mapping. Since we were mainly interested in normal strain, we calculated the hydrostatic normal strain^[^ ^43^ ^]^ after extracting the atomic positions using our AtomID model and the other methods. We selected a low‐quality STEM image of a defect‐free area of MoS2 for testing, as confirmed by a high‐quality STEM image of the same location. By comparing the strain mapping results calculated by the different models and by GPA strain analysis, we found that our AtomID‐Net (−0.00141 ± 0.0265) had the results closest to GPA (−0.0178 ± 0.0132), demonstrating our model's good performance on noisy images (**Figure** **6**).

### 3.5 Time Consumption

We also built modified versions of our model by replacing the NMS module with nonlearning detection algorithms, including Laplacian of Gaussian (LoG), Difference of Gaussian (DoG), and Histogram of Oriented Gradients (HOGs) from scikit image.^[^ ^44^ ^]^ The maximum sigmas were set to the root square of the average spacing, with an upper bound of 10, to match the spacing parameter in NMS. The thresholds were set to 0.1, 0.1, 0.004 respectively, while parameters were kept at their default values. The results are shown in **Table** **2**.

**Table 2:** Testing results on atom detection

Metric	[CD]	Jaccard	F1	Time
LoG	2.252 ± 1.036	0.951 ± 0.039	0.978 ± 0.023	727.017 ±140.831 ms pic^−1^
DoG	2.320 ± 1.160	0.963 ± 0.0307	0.970 ± 0.036	474.637 ± 139.721 ms pic^−1^
HoG	4.074 ± 1.336	0.972 ± 0.026	0.939 ± 0.082	1.192 ± 0.224 s pic^−1^
Ours(cpu)	2.373 ± 1.176	0.967 ± 0.025	0.964 ± 0.039	231.903 ± 44.965 ms pic^−1^
Ours(gpu)				52.854 ± 2.399 ms pic^−1^

Our proposed detection module without parameter tuning outperformed the strict algorithms that required fine tuning. Moreover, our method had shorter latency and can work in real time.

## 4 Conclusion

Accurately detecting atoms or atomic columns in multiscale, noisy TEM images are essential for material characterization and understanding of material properties. In this article, we introduce AtomID‐Net, a deep neural network, for atom detection in low‐SNR STEM images. We also propose a new labeling method for noisy TEM images, which uses the high‐quality images as references to annotate the noisy ones taken at the same sample position. Our model outperforms pure 2D template fitting methods for atom detection in noisy images, and its flexibility allows it to perform well at multiple scales compared to nonlearning methods. Furthermore, our model excels in strain calculation, demonstrating its high accuracy in atomic position identification. In future work, we plan to reformulate the model to leverage prior knowledge for improved performance.

## 5 Experimental Section

5.1

5.1.1

##### MoS_2_ Preparation

The monolayer MoS_2_ was grown via atmospheric pressure chemical vapor deposition (CVD) on (001)‐cut fluorophlogopite mica within 2‐inch CVD furnace. The mica was placed face down above a quartz boat containing 3 mg of molybdenum trioxide (Innochem, 99%) with another crucible containing 120 mg of sulfur (Aldrich, 99.998%) located at the upstream. The growth recipe was set 350 s.c.c.m argon for 10 min; then, gas flow was adjusted to 50 s.c.c.m argon, heated to 800 °C with a ramping rate of 16 °C min^−1^, and kept at 800 °C for 5 min, while the upper side was kept at 180 °C during the synthesis. After the reaction, the furnace naturally cooled to room temperature.

##### ReS_2_ Preparation

Monolayer (1L) rhenium disulfide (ReS_2_) was grown via atmospheric pressure CVD on a single‐side polished c‐face sapphire substrate. Sulfur powder (Aldrich, 99.998%) was put in the upstream zone while ammonium perrhenate (NH_4_ReO_4_) (Aldrich, 99.999%) was put in the downstream zone as precursors. The polished side of c‐face sapphire substrate was toward the top of the Re source and the size was 1 cm × 1 cm. The growth recipe was: set 300 s.c.c.m argon for 10 min; then, gas flow was adjusted to 80 s.c.c.m argon. Then the upstream zone was heated to 200 °C and the downstream zone to 850 °C. The heat process took 30 min and held for 10 min.

##### TEM Sample Preparation

To transfer the as‐grown MoS_2_ onto Cu quantifoil grid, MoS_2_ was coated with a layer of poly(meththyl methacrylate) (PMMA) (A4) by spin coating of 3000 rpm for 1 min, followed by baking at 100 °C for 5 min. The process was repeated for two layers of PMMA coating. Next, the PMMA/MoS_2_ on mica was immersed in deionized (DI) water for 5 min and then detached by gentle pulling. Subsequently, we fished out the floating PMMA/MoS_2_ film with a grid, placed the grid on a hot plate at 60 °C for 10 min, and stored under vacuum for 12 h. The PMMA was then removed by rinsing acetone, IPA, and DI water. After that, the grid was annealed at 200 °C for 1100 min in order to remove residues of hydrocarbons on the surface.

##### TEM Experiments

Experimental HAADF‐STEM imaging was performed using an aberration‐corrected TEM (Spectra 300 TEM) operated at 300 kV, and the electron beam current was around 10–20 pA. The CL aperture during image capture was 115 mm and the collection angle was 45 to 180 mrad; the dwell time was set to 10 μs and the original images were 1024 × 1024 pixels.

## Conflict of Interest

The authors declare no conflict of interest.

## Supporting information

Supplementary Material

## Data Availability

The data that support the findings of this study are available from the corresponding author upon reasonable request.
