# Supplementary material for: A Multiscale Deep‐Learning Model for Atom Identification from Low‐Signal‐to‐Noise‐Ratio Transmission Electron Microscopy Images
Source: Small Sci. 2023 Jun 11;3(8):2300031. doi: 10.1002/smsc.202300031 (PMC11935788; doi:10.1002/smsc.202300031)
Supplement: Supplementary file 1 — Supplementary Material [file SMSC-3-2300031-s001.pdf]

Supporting Information

# A Multiscale Deep-Learning Model for Atom Identification from low-Signal-toNoise-Ratio Transmission Electron Microscopy Images

Authors *Yanyu Lin*[1+], *Zhangyuan Yan*[2+], *Chi Shing Tsang*[2], *Lok Wing Wong*[2,3], *Xiaodong Zheng*[2,3], *Fangyuan Zheng*[2], *Jiong Zhao*[2,3]*, *Ke Chen*[1,4]*

Affiliations

1, School of Future Technology, South China University of Technology, Guangzhou 510641, China

2, Department of Applied Physics, The Hong Kong Polytechnic University, Kowloon, Hong Kong, China.

3, Polytechnic University of Hong Kong Shenzhen Research Institute, Shenzhen, China

4, School of Electronic and Information Engineering, South China University of Technology, Guangzhou 510641, China

*E-mail: jiong.zhao@polyu.edu.hk,
chenk@scut.edu.cn

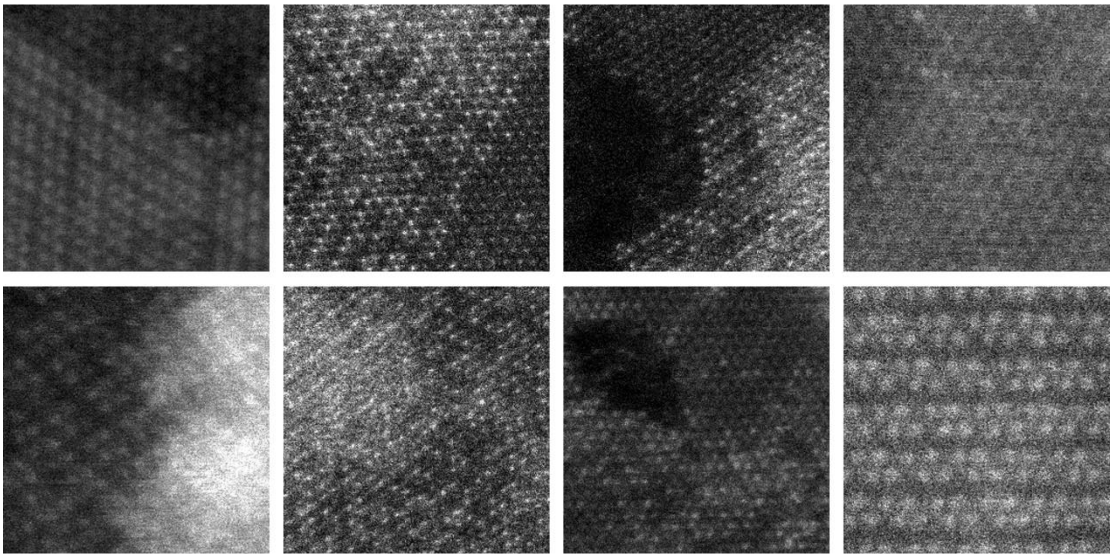

**Figure S1. Training set of real HAADF images.**

## Note 1. Parameters of other methods

### 1.1 Findfoci

To filter out background noise, we set the absolute background parameters in the range of 0.1-0.3, and then we apply Gaussian blur to the image to reduce spurious peaks and smooth the image. The value of Gaussian blur is set between 0.8 and 2. We set the minimum size of the peak as 10, but we adjust this parameter for images with different magnifications to prevent atomic peaks from splitting into smaller ones.[1]

### 1.2 Atomap

We use a peak finding algorithm from the Python package scikit-image in Atomap, which requires the smallest peak separation. To find the optimal pixel separation, we use the Atomap function *get_feature_separation()*. For our test set, the separation range is 4 to 10, and it changes with different magnifications of the image.[2]

### 1.3 AtomSegNet

AtomSegNet offers 15 different models for atomic position detection. After testing on our validation set, we found that the model *denoise&gremoval&superres* performed the best. Therefore, we mostly used this model to test the images in our validation set, but we also used the model *gaussianMask+* sometimes. We use the parameter "Up sample by 2" to enlarge the image by two times, which helps the model detect images with dense arrays of atoms. We sometimes use this parameter for low magnification images.[3]

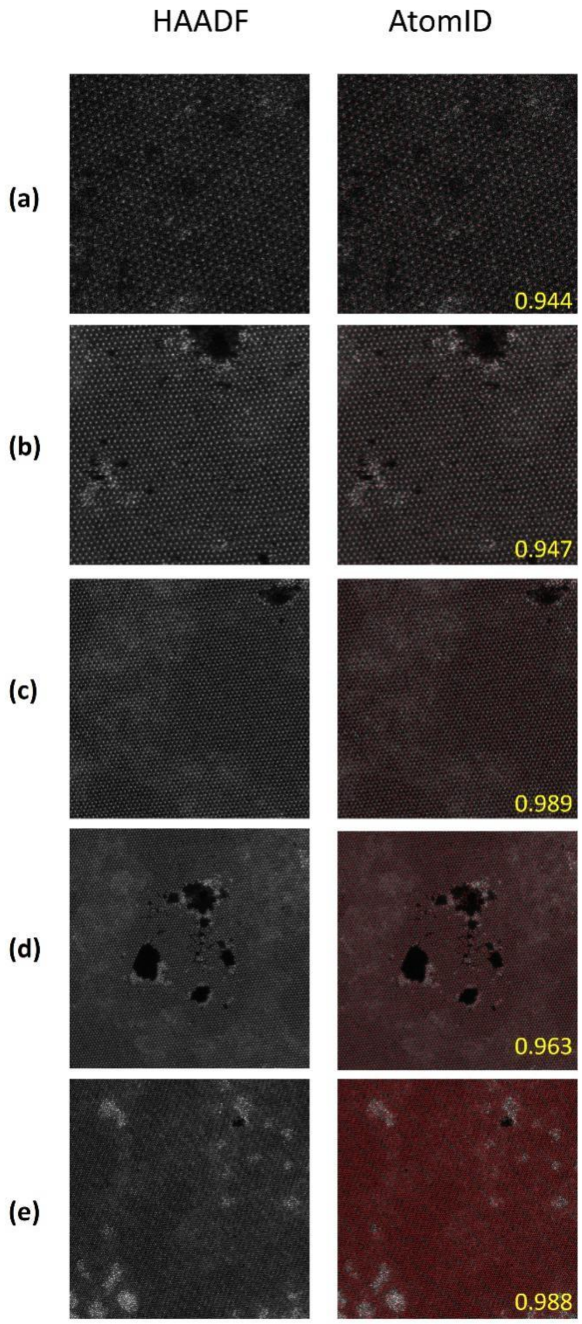


**Figure S2. AtomID model's results for HAADF images with different scale. This model is flexible and is not limited to a fixed scale of atoms. F1-score is shown in yellow. Atomic scale: (a) 8.99nm × 8.99nm; (b) 12.7nm × 12.7nm; (c)18.0nm × 18.0nm; (d) 25.4nm × 25.4nm; (e) 36.0nm × 36.0nm**

References:

1. Herbert, A. D., ImageJ FindFoci Plugins. *FindFoci. Nov* **2014,** *18*.

2. Lin, R.; Zhang, R.; Wang, C.; Yang, X.-Q.; Xin, H. L., TEMImageNet training library and AtomSegNet deep-learning models for high-precision atom segmentation, localization, denoising, and deblurring of atomic-resolution images. *Scientific reports* **2021,** *11* (1), 5386.

3. Nord, M.; Vullum, P. E.; MacLaren, I.; Tybell, T.; Holmestad, R., Atomap: a new software tool for the automated analysis of atomic resolution images using two-dimensional Gaussian fitting. *Advanced structural and chemical imaging* **2017,** *3*, 1-12.
